# Casein sIgE as the most accurate predictor for heated milk tolerance in Finnish children

**DOI:** 10.1111/pai.70152

**Published:** 2025-07-18

**Authors:** Otso Nieminen, Kati Palosuo, Mika J. Mäkelä

**Affiliations:** ^1^ Department of Allergology University of Helsinki and Helsinki University Hospital Helsinki Finland

**Keywords:** casein, children, cow's milk allergy, cutoff, heated milk, oral food challenge, skin prick test

## Abstract

**Background:**

Up to 83% of children with cow's milk allergy (CMA) tolerate baked milk, but research on heated milk (HM) tolerance and allergen‐specific IgE (sIgE) cutoff values predicting oral food challenge (OFC) outcomes are lacking. We hypothesized that casein sIgE is the best predictor for HM tolerance.

**Methods:**

Finnish children suspected of having IgE‐mediated CMA were recruited. sIgE levels to milk, casein, alpha‐lactalbumin, beta‐lactoglobulin, and bovine serum albumin were measured alongside skin prick tests (SPT) to fresh milk (FM) and HM. The diagnosis was confirmed with an FM OFC. IgE‐sensitized children with a positive FM OFC underwent an HM OFC with extensively heated milk in rice porridge. Cutoff values to predict HM OFC outcome were determined, reaction severity analyzed, and effect of age investigated.

**Results:**

Of the 158 children (median age: 2.59 years) undergoing FM OFC, 127 proceeded to an HM OFC. Of these, 77 (61%) demonstrated HM tolerance. Casein sIgE emerged as the best predictor for the HM OFC outcome (AUC 0.786), with a 95% sensitivity cutoff value of <0.54 kU/L and a 95% specificity cutoff value of >14.1 kU/L. Most HM OFC reactions were mild (66%), but reaction severity was unpredictable. Casein sIgE, milk sIgE, and HM SPT showed greater accuracy in ≤3‐year‐old children.

**Conclusion:**

Casein sIgE cutoffs <0.54 and >14.1 kU/L can help identify children with a high or low likelihood of HM tolerance. This approach may reduce unnecessary OFCs in high‐risk patients and guide safe dietary advancement on a milk ladder in low‐risk cases.

**Key message**

Most cow's milk allergic children tolerate baked milk, but cutoff values to predict heated milk allergy and tolerance are lacking. We confirmed the role of casein sIgE as the best predictor for this purpose. Based on a carefully screened oral food challenge confirmed IgE‐mediated cow's milk allergic population of Finnish children, we established cutoff values to reduce the need for oral food challenges. This will benefit clinicians treating this most common food allergy of children.

## 1 INTRODUCTION

Baked milk (BM) tolerance is commonly considered a precursor for the outgrowth of cow's milk allergy (CMA).^1^, ^2^, ^3^, ^4^ The majority of cow's milk allergic children tolerate BM, with estimates ranging between 62.5% and 83%,^4^, ^5^, ^6^, ^7^, ^8^, ^9^ although a lower prevalence has been reported in a high‐risk population.^10^ It has also been suggested that BM tolerance represents a transient phenotype of CMA compared to BM intolerance.^11^, ^12^ Other studies have proposed that regular use of BM could accelerate tolerance development to fresh milk (FM),^3^, ^4^ with the more recent studies strengthening this view.^13^, ^14^, ^15^ This possibility for an intervention to speed up CMA outgrowth, together with the claimed high‐severity phenotype of BM intolerance^3^, ^6^, ^16^ makes it important to correctly diagnose a child's BM allergy status.

Considering the effect of heating on the proteins in cow's milk, caseins (Bos d 8) are highly heat‐stabile, whereas whey proteins, such as alpha‐lactalbumin (Bos d 4) and beta‐lactoglobulin (Bos d 5), are heat‐labile and lose allergenicity during extensive heating.^17^ Most BM allergy studies have used baked products, such as muffins, where milk proteins are embedded in a matrix that contains wheat and possibly eggs.^2^, ^4^, ^5^, ^6^, ^18^, ^19^, ^20^, ^21^, ^22^ It is well known that baking milk with such a matrix reduces its allergenicity.^17^, ^23^, ^24^ However, studies on extensively heated milk (HM) outside a baked matrix are lacking. To answer this need, we used HM (heated in 175**°**C for 90 min) in a rice‐based matrix in our oral food challenges (OFC) to assess HM tolerance.

We hypothesized that casein‐specific IgE (sIgE) would be the best predictor for HM tolerance due to its heat stability.^17^ Our objectives were to establish clinically useful cutoff values for sIgE and skin prick tests (SPT) to predict HM OFC outcomes, to examine the effect of age on these cutoff values, and to explore whether reaction severity could be predicted.

## 2 METHODS

### 2.1 Primary and secondary outcomes

The primary outcome was to establish clinically useful cutoff values for casein sIgE to identify HM‐reactive from tolerant patients. Secondary outcomes included assessing the diagnostic usefulness of sIgE to milk, alpha‐lactalbumin, beta‐lactoglobulin, and BSA; evaluating the additional value of FM SPT and HM SPT in diagnostics; examining reaction severity and cumulative dose in HM OFCs; and investigating the impact of age on the cutoffs, HM tolerance rate, and reaction severity. Furthermore, the relationship between reaction severity and cumulative eliciting dose in positive HM OFCs and the preceding FM OFCs was examined.

### 2.2 Power calculations

Using data from a previous study,^4^ we calculated that 128 patients were required for sufficient power to reach the primary outcome (alpha‐error 0.05, beta‐error 0.8).

### 2.3 Inclusion and exclusion criteria

Patient recruitment took place between the years 2021–2024 at Helsinki University Skin and Allergy Hospital. Inclusion criteria were ages between 1 and 18 years, clinical suspicion of CMA confirmed by an OFC, and sensitization to milk measured by FM SPT, HM SPT, or sIgE to milk, casein, alpha‐lactalbumin, or beta‐lactoglobulin within 6 months of both the FM and the HM OFC. Exclusion criteria were a severe anaphylactic reaction to milk within 1 year or in the initial FM OFC; uncontrolled asthma or atopic dermatitis; severe cardiac or pulmonary disease; a malignant tumor; beta‐blocker medication; an active immunological disease; mastocytosis; severe fear of needles; and lack of compliance with the research protocol.

### 2.4 Ethical approval

The Helsinki University Hospital of Children and Adolescents Ethics Committee gave ethics approval for the study. Each child ≥6 years of age and one of the guardians signed a written consent to participate in the study. The Declaration of Helsinki was followed throughout the study.

### 2.5 Allergy testing

sIgE to milk (f2), casein (Bos d 8), alpha‐lactalbumin (Bos d 4), beta‐lactoglobulin (Bos d 5), and bovine serum albumin (BSA, Bos d 6) was measured from venous blood samples by ImmunoCAP (Thermo Fisher, Uppsala, Sweden). SPTs (skim milk, Arla, Sipoo, Finland) were measured for FM and HM (milk boiled for 15 min), with a wheal size of <3.0 mm being considered negative and treated as null in the analysis.

### 2.6 Oral food challenges

Diagnosis of CMA was confirmed with an open FM OFC (pasteurized, defatted cow's milk) following a previously published protocol.^25^ Children with a positive challenge reaction were subjected to an HM OFC with extensively heated rice porridge (cooked in the oven at 175**°**C for 90 min, recipe in supplements). Five doses were administered at 30‐minute intervals, the amount of milk protein in the doses being 15, 60, 310, 1550, and 3100 mg, adding up to a cumulative dose of 5035 mg if all doses were given. ≤2‐year‐old children, however, did not receive the last dose due to the large quantity of porridge in the serving.

The challenges were interpreted as positive according to the PRACTALL consensus criteria^26^ and reaction severity was assessed with a modified version of Sampson's severity scoring (mSSS)^27^ and a modified version of Hourihane's severity scoring (mHSS).^28^ After a negative challenge, HM was freed in the diet. In the case of a mild reaction (mSSS^27^ grade 1–2) from a 1550 or 3100 mg dose, use of HM was instructed at home 3–4 times a week, beginning from 10 and 50 mL, respectively, and increasing 10 mL per month if tolerated. Recipes on how to use HM/BM for cooking at home were offered to the families. In other cases, complete avoidance of milk was instructed.

### 2.7 Age group comparisons

The following were compared between ≤3‐year‐old and >3‐year‐old children: HM tolerance rates; the predictive performance of sIgE and SPT for HM OFC outcomes, including age‐specific cutoff values; OFC reaction severity; and cumulative doses. The cutoff of 3 years was chosen as the tolerance development rate has been shown to slow down in older children.^29^

### 2.8 Statistical analyses

Statistical analyses were performed with SPSS Inc. (Chicago, IL) version 28 software. SPSS, Microsoft Excel (version 2412) and Inkscape software (version 1.2.2) were used for figure making. Normality of data was tested with Kolmogorov–Smirnov and Shapiro–Wilk tests. Due to data's non‐normality, Mann–Whitney *U* test and Kruskal–Wallis test were used for ordinal and continuous variables, and chi–square test was used to test for differences in categorical variables. Wilcoxon signed ranks test and McNemar's test were used to compare related samples. Cutoff values for 95% specificity and 95% sensitivity were determined with receiver operating characteristic (ROC) curves, and positive predictive values (PPV), negative predictive values (NPV), and positive and negative likelihood ratios (LR+, LR–) were calculated. Optimal cutoff points were determined using Youden's index. The 95% specificity cutoff value was chosen as predictive of HM intolerance, whereas the 95% sensitivity cutoff value was deemed predictive of tolerance.

## 3 RESULTS

### 3.1 Study population and HM tolerance rate

**FIGURE 1:**
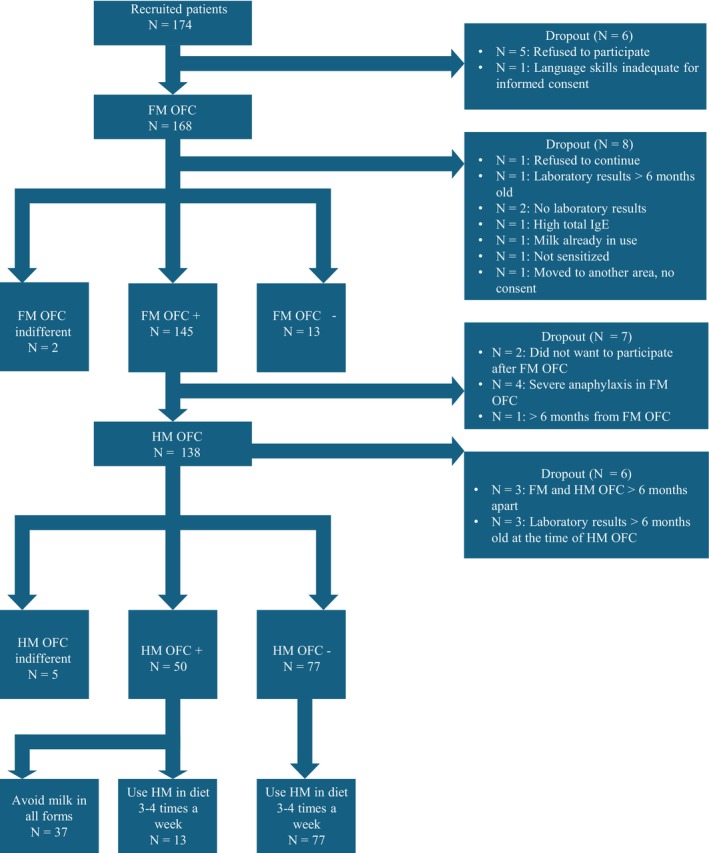
The study protocol and flowchart. Presented are the number of children participating in each step of the study. The number of dropouts and the reasons for dropping out are provided on the right side of the figure.

We recruited 174 children, of whom 168 participated in the study and underwent an FM OFC (Figure 1). Of these 168 children, 158 (94.0%) qualified for the study (median age: 2.59 years, interquartile range (IQR): 1.51–5.33 years). 145 (91.8%) of the 158 FM OFCs were positive, and 138 children continued to have an HM OFC. 127 children (median age: 2.84 years, IQR: 1.67–5.39 years) with HM OFCs remained qualified for analysis after excluding 6 dropouts and 5 indifferent OFC results. 50 HM OFCs were positive, and 77 were negative, establishing an HM tolerance rate of 61%.

### 3.2 Baseline characteristics

The baseline characteristics, median sIgE values, median SPT wheal sizes, and median cumulative doses in the HM OFC positive and negative groups appear in Table 1. HM tolerance was associated with younger age (*p* = .05). sIgEs to milk, casein, and alpha‐lactalbumin differed significantly between the two groups. Casein sIgE levels were higher in children who were allergic to HM, with a median of 4.56 kU/L in the HM OFC–positive group compared to 0.71 kU/L in the HM OFC–negative group. The respective values for milk sIgE were 9.28 kU/L and 3.64 kU/L. Likewise, FM SPT and HM SPT wheal sizes were significantly larger in the HM OFC–positive group (medians 9.0 and 8.0 mm, respectively) compared to the HM OFC–negative group (medians 7.0 and 5.0 mm, respectively).

**TABLE 1:** Baseline characteristics, median values of SPTs and sIgEs, and age‐related cumulative doses according to HM OFC status.

	HM OFC– (*N* = 77)	HM OFC+ (*N* = 50)	*p*‐Values
Median sIgE, kU/L (IQR)			
Milk (f2)	3.64 (1.29–10.5)	9.28 (3.47–19.95)	**<.001** *
Casein (Bos d 8)	0.71 (0.20–2.97)	4.56 (1.89–9.89)	**<.001** *
Alpha‐lactalbumin (Bos d 4)	1.40 (0.25–4.75)	3.47 (1.20–13.50)	.**017** *
Beta‐lactoglobulin (Bos d 5)	1.07 (0.31–4.30)	2.38 (0.61–6.92)	.066
BSA (Bos d 6)	0.01 (0.00–0.17)	0.01 (0.00–0.12)	.785
Median SPT wheal size, mm (IQR)			
Fresh milk	7.0 (5.3–9.0)	9.0 (6.0–12.0)	.**029** *
Heated milk	5.0 (4.0–6.0)	8.0 (5.0–8.0)	**<.001** *
Egg	4.0 (0.0–5.0)	3.5 (0.0–6.0)	.762
Wheat	0.0 (0.0–0.0)	0.0 (0.0–3.0)	.372
Gliadin	0.0 (0.0–0.0)	0.0 (0.0–0.0)	1.000
Peanut	2.5 (0.0–5.8)	5.0 (0.0–11.0)	.**003** *
Cashew nut	5.0 (0.0–9.0)	6.0 (0.0–9.3)	.959
Baseline characteristics			
Sex, male, *N* (%)	46 (59.7)	31 (62.0)	.799
Median age (years) at HM OFC (IQR)	2.44 (1.50–5.20)	3.30 (2.18–6.83)	.**05** *
Atopic dermatitis, *N* (%)^a^	9 (11.7)/33 (42.9)/35 (45.5)	2 (4.0)/15 (30.0)/33 (66.0)	.055
Asthma, *N* (%)^a^	66 (85.7)/4 (5.2)/7 (9.1)	32 (64.0)/4 (8.0)/14 (28.0)	.**012** *
Pollen allergy, *N* (%)^a^	48 (62.3)/3 (3.9)/26 (33.8)	23 (46.0)/1 (2.0)/26 (52.0)	.119
Animal dust allergy, *N* (%)^a^	54 (70.1)/5 (6.5)/18 (23.4)	30 (60.0)/–/20 (40.0)	.**038** *
Other food allergies, median (IQR)	1.0 (0.5–2.0)	1.0 (0.75–2.0)	.482
Atopic dermatitis in family, *N* (%)	47 (61.0)	38 (76.0)	.08
Asthma in family, *N* (%)	21 (27.3)	19 (38.0)	.221
Allergies in family, *N* (%)	60 (77.9)	41 (82.0)	.578
Tobacco smoking in family, *N* (%)	6 (7.8)	4 (8.0)	.966
Median HM OFC cumulative dose, mg			
≤3‐year‐old (IQR)	1935 (1935–2090)	1160 (385–1950)	NA
>3‐year‐old (IQR)	5035 (3513–5035)	385 (135–1935)	NA

*Note*: Baseline characteristics were determined through questionnaires. *p*‐values refer to statistical comparison between HM OFC+ and HM OFC– groups. Statistical testing for cumulative dose is not applicable, as all doses are consumed in a negative challenge. Missing information: asthma in family from 1 patient, SPT from 5 patients. Other food allergies screened for: egg, any nut, fish, wheat, soy.

Abbreviations: BSA, bovine serum albumin; HM OFC, heated milk oral food challenge; HM OFC–, negative HM OFC; HM OFC+, positive HM OFC; IQR, interquartile range; kU/L, kilounits per liter; mm, millimeter; NA, not applicable; sIgE, specific immunoglobulin E.

* Bold Denotes a statistically significant difference (*p* < .05).

^a^ Never/previously/currently.

### 3.3 ROC analysis and cutoff values

**FIGURE 2:**
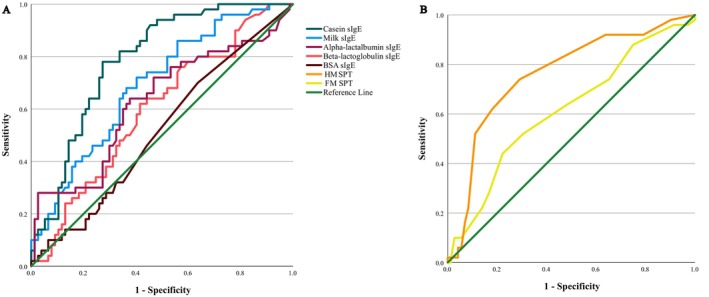
The receiver operating characteristic curves to predict the HM oral food challenge result in the whole study population for casein, milk, alpha‐lactalbumin, beta‐lactoglobulin, and BSA sIgE (A, *N* = 127) and FM and HM SPT (B, *N* = 122). BSA, Bovine serum albumin; FM, Fresh milk; HM, Heated milk; sIgE, Specific immunoglobulin E; SPT, Skin prick test.

Casein sIgE was the best predictor for HM OFC outcome with an area under the curve (AUC) of 0.786, followed closely by the HM SPT (AUC 0.762) (Figure 2, Table 2). However, all the predictors studied had overlapping 95% confidence intervals (CI) for AUCs. The 95% specificity cutoff point predictive of a positive HM OFC was reached at >14.1 kU/L for casein sIgE (sensitivity 18.0%), at >33.9 kU/L for milk sIgE (sensitivity 14.0%), and at >11.5 mm for HM SPT (sensitivity 6.0%). The 95% sensitivity predictive of a negative HM OFC was reached at <0.54 kU/L for casein sIgE (specificity 45.5%) and at <1.46 kU/L for milk sIgE (specificity 27.8%). For HM SPT, the highest sensitivity achievable was 92.0%, which was reached at <3.5 mm (specificity 20.8%). Table 2 summarizes the AUCs, cutoff points, and corresponding specificities, sensitivities, PPVs, NPVs, LRs+, and LRs–, and presents the optimal cutoff values.

**TABLE 2:** Cutoff values for casein, milk, and alpha‐lactalbumin sIgE (*N* = 127) and HM SPT and FM SPT (*N* = 122) to predict HM OFC outcome. Cutoff points closest to 95% specificity and 95% sensitivity were determined, and optimal cutoff values were chosen based on maximal Youden's index.

	AUC (95% CI)	Cutoff (kU/L, mm)	Specificity (%)	Sensitivity (%)	PPV (%)	NPV (%)	LR+	LR–	*p*‐Value
95% specificity cutoff									
Casein sIgE	0.786 (0.708–0.864)	14.1	94.8	18.0	69.2	64.0	3.47	0.86	<0.001*
Milk sIgE	0.690 (0.598–0.782)	33.9	94.8	14.0	63.6	62.9	2.70	0.91	<0.001*
Alpha‐lactalbumin sIgE	0.626 (0.523–0.728)	9.85	94.8	28.0	77.8	67.0	5.39	0.76	0.017*
FM SPT	0.616 (0.514–0.718)	13.5	94.4	10.0	55.6	60.2	1.8	0.95	0.03*
HM SPT	0.762 (0.673–0.851)	11.5	95.8	6.0	50.0	59.5	1.44	0.98	<0.001*
95% sensitivity cutoff									
Casein sIgE	0.786 (0.708–0.864)	0.54	45.5	96.0	53.3	94.6	1.76	0.088	<0.001*
Milk sIgE	0.690 (0.598–0.782)	1.46	27.8	96.0	46.2	91.3	1.32	0.15	<0.001*
Alpha‐lactalbumin sIgE	0.626 (0.523–0.728)	0.03	5.2	94.0	39.2	57.1	0.99	1.16	0.017*
FM SPT	0.616 (0.514–0.718)	4.5	8.3	96.0	42.1	75.0	1.05	0.48	0.03*
HM SPT	0.762 (0.673–0.851)	3.5	20.8	92.0	44.7	78.9	1.16	0.38	<0.001*
Optimal cutoff									
Casein sIgE	0.786 (0.708–0.864)	1.74	72.7	78.0	65.0	83.6	2.86	0.30	<0.001*
Milk sIgE	0.690 (0.598–0.782)	4.92	59.7	72.0	53.7	76.7	1.79	0.47	<0.001*
Alpha‐lactalbumin sIgE	0.626 (0.523–0.728)	2.54	62.3	64.0	52.5	72.7	1.70	0.58	0.017*
FM SPT	0.616 (0.514–0.718)	9.5	77.8	44.0	57.9	66.7	1.98	0.72	0.03*
HM SPT	0.762 (0.673–0.851)	5.5	70.8	74.0	63.8	79.7	2.54	0.37	<0.001*

*Note*: Cutoffs are in kU/L for sIgE and in mm for SPTs. Beta‐lactoglobulin (*p* = .066) and BSA (*p* = .790) were similar between the groups (Table S1A). 95% sensitivity could not be reached with HM SPT.

Abbreviations: AUC, area under the curve; CI, confidence interval; FM, fresh milk; HM, heated milk; kU/L, kilounits per liter; LR–, negative likelihood ratio; LR+, positive likelihood ratio; mm, millimeter; NPV, negative predictive value; OFC, oral food challenge; PPV, positive predictive value; sIgE, specific immunoglobulin E; SPT, skin prick test; *, statistically significant difference (*p* < .05).

### 3.4 Cumulative dose and reaction severity

**FIGURE 3:**
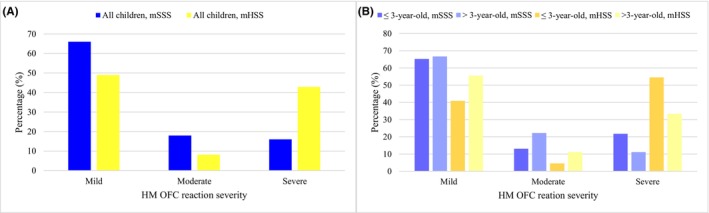
The severity of positive HM OFC reactions scored by mSSS^27^ and mHSS^28^ in the whole population (A, *N* = 50) and separately in the groups of ≤3‐year‐old (*N* = 23) and >3‐year‐old children (*N* = 27) (B). One reaction could not be scored with mHSS^28^ as it did not recognize diarrhea as a symptom. HM OFC, Heated milk oral food challenge; mSSS^27^, Modified Sampson's severity scoring; mHSS^28^, Modified Hourihane's severity scoring.

Among the 50 positive HM OFCs, the median cumulative eliciting dose was 941 mg (IQR: 323–1935 mg). Using the mSSS^27^, most reactions were mild (66%), with nine (18%) classified as moderate and eight (16%) as severe. However, with mHSS^28^ the proportion of severe reactions increased (Figure 3A). Five (10%) of the HM OFC reactions were considered anaphylactic as defined by Sampson et al.,^30^ and 10 (20%) patients received epinephrine, one of them twice for the same reaction. There were no moderate‐to‐severe (mSSS^27^) or anaphylactic reactions if casein sIgE was <0.54 kU/L. The HM cumulative dose or sIgE levels did not differ between mild, moderate, or severe reactions regardless of whether mSSS^27^ or mHSS^28^ was used for grading. However, with mSSS^27^ there was a trend towards significance with *p*‐values of .062 for milk sIgE, .070 for alpha‐lactalbumin, and .051 for beta‐lactoglobulin. With regards to SPT wheal size, no difference was found using mSSS^27^, while with mHSS^28^ the HM SPT had a *p*‐value of .043 for the difference between mild and moderate reactions. With mHSS^28^, severely reacting children were younger than mildly reacting children (*p* = .039, Bonferroni corrected), whereas mSSS^27^ showed similar ages in all reaction severity categories (*p* = .531).

A higher cumulative dose in the FM OFC was associated with a negative HM OFC outcome (*p* = .018), whereas FM OFC reaction severity and HM OFC outcome were not related (*p* = .375). Regarding the positive HM OFCs (*N* = 50), FM OFC reaction severity (mSSS^27^) did not affect the likelihood of a moderate‐to‐severe reaction to HM (*p* = .359, Figure S1A). However, the cumulative eliciting doses were higher compared to the preceding FM OFCs (*p* < .001, Figure S1B).

### 3.5 Effect of age and age specific cutoff values

The tolerance rate was 66% in the younger (≤3‐year‐old, *N* = 68) and 54% in the older (>3‐year‐old, *N* = 59) age group, with no significant difference in the chi‐square test (*p* = .170). In both age groups, milk and casein sIgE levels were significantly higher (Table S1B,C) and HM SPT wheal sizes were larger (Table S2) in HM OFC‐positive children compared to HM tolerant children. sIgE levels to alpha‐lactalbumin, beta‐lactoglobulin, or BSA (Table S1B,C), as well as FM SPT wheal sizes (Table S2), showed no significant differences.

**FIGURE 4:**
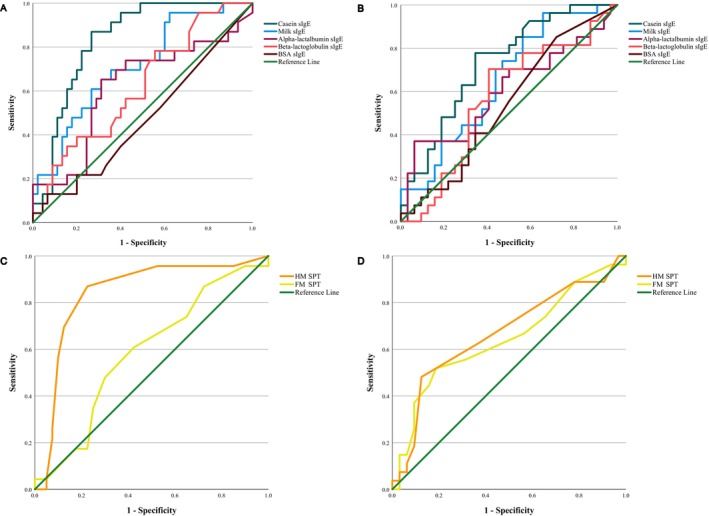
The receiver operating characteristic curves to predict the HM oral food challenge result for casein, milk, alpha‐lactalbumin, beta‐lactoglobulin, and BSA sIgE in ≤3‐year‐old (A, *N* = 68) and >3‐year‐old children (B, *N* = 59), and for FM and HM SPT in ≤3‐year‐old (C, *N* = 63) and >3‐year‐old children (D, *N* = 59). BSA, Bovine serum albumin; FM, Fresh milk; HM, Heated milk; sIgE, Specific immunoglobulin E; SPT, Skin prick test.

Age‐specific cutoff values for sIgE (Table S1B,C) and SPT (Table S2) corresponding to 95% specificity, 95% sensitivity, and the optimal cutoff point were calculated, and corresponding ROC curves appear in Figure 4. For ≤3‐year‐old children, the casein sIgE cutoff value corresponding to 95% specificity was >14.1 kU/L (sensitivity 13.0%), while for the >3‐year‐old children, it was >20.7 kU/L (sensitivity 18.5%). The 95% sensitivity was reached at <0.81 kU/L (specificity 60.0%) in the younger and at <0.33 kU/L (specificity 31.2%) in the older age group. For HM SPT, the cutoff value <4.5 mm corresponded to 95% sensitivity and 47.5% specificity in the ≤3‐year‐old age group, while in the group of >3‐year‐old children, 95% sensitivity could not be reached. There were no differences between the age groups in terms of reaction severity with either mSSS^27^ (*p* = .481) or mHSS^28^ (*p* = .295) (Figure 3B). There were no moderate‐to‐severe or anaphylactic reactions at casein sIgE level <0.81 kU/L in the younger age group. Furthermore, cumulative doses among HM OFC positive children were similar in both groups (*p* = .397).

## 4 DISCUSSION

We investigated heated cow's milk reactivity and tolerance in a highly selected population of Finnish children (median age: 2.84) with OFC‐confirmed, IgE‐mediated CMA referred to a tertiary hospital. As the primary outcome, aligning with our initial hypothesis, casein sIgE emerged as the best sIgE predictor of HM OFC outcome (Table 2). As for secondary outcomes, HM SPT performed well in predicting HM tolerance (Table 2). Overall, the HM tolerance rate of 61% was lower than previously reported,^4^, ^5^, ^6^, ^7^, ^8^, ^9^ with HM tolerance being associated with younger age (*p* = .05) and a higher FM OFC cumulative dose (*p* = .018). Moreover, the accuracy of casein and milk sIgE (Table S1B,C) and HM SPT (Table S2) was higher in children aged ≤3 years compared to older children. Reaction severity remained unpredictable, but cumulative doses were higher in positive HM OFCs compared to the preceding FM OFCs.

Casein sIgE was the best predictor for HM OFC outcome with an AUC of 0.786, although 95% CIs overlapped with the AUCs of other studied milk allergens (Table 2, Figure 2). Despite our results, controversy on the usefulness of sIgE to predict BM OFC outcome remains evident.^6^, ^10^, ^18^, ^19^, ^20^, ^22^, ^31^, ^32^, ^33^ Most studies, however, agree that higher casein sIgE levels are associated with BM intolerance.^6^, ^19^, ^20^, ^31^, ^32^, ^33^ In our study, a casein sIgE cutoff value of >14.1 kU/L provided 95% specificity with 18% sensitivity. Previously, casein sIgE has been purported as the best predictor for BM reactivity (AUC 0.806),^31^ with cutoff values around 20 kU/L yielding 82%–95% specificity and 30%–50% sensitivity,^19^, ^31^ although a much lower cutoff value of >3.01 kU/L (AUC 0.755, 84% specificity, 80% sensitivity) has been reported in infants.^20^ The lower sensitivities in our study are probably explained by the strict inclusion criteria and the use of HM instead of BM, but differences in age, ethnicities, and the level of sensitization may also play a role.

For predicting HM tolerance, a casein sIgE cutoff value of <0.54 kU/L provided 95% sensitivity with an NPV of 94.6%, demonstrating its reliability in ruling out HM reactivity (Table 2). Our findings are in line with previous reports of cutoffs <1.00 kU/L (100% sensitivity, 18% specificity)^19^ and <0.94 kU/L (95% sensitivity, 32% specificity).^31^ For milk sIgE, our 95% sensitivity cutoff of <1.46 kU/L was comparable to the <1 kU/L cutoff reported by Agyemang et al.,^21^ but lower than the commonly used <5 kU/L threshold associated with a 90% BM tolerance rate.^6^

HM SPT had a fair AUC of 0.762 in predicting HM OFC outcome (Table 2, Figure 2B) but the role of SPTs in BM allergy diagnostics remains contradictory.^5^, ^6^, ^8^, ^18^, ^19^, ^22^ For milk SPT, a cutoff of ≤5 mm has been proposed (AUC 0.828, specificity 63.6%, sensitivity 78.9%),^8^ while for muffin slurry SPT, cutoff values <3.0 mm (100% sensitivity) and >8.0 mm (83% specificity) have been suggested.^19^ Compared to our results, these cutoffs appear to perform better, possibly due to similar factors as with the sIgE outcomes, but also due to additional factors such as the manufacturing process of extracts used.

The severity of reactions in positive HM OFCs remained unpredictable as previously reported for CMA.^34^ Levels of sIgE, SPT wheal sizes, and the HM OFC cumulative dose were not reliably associated with reaction severity. Severely reacting children tended to be younger than mildly reacting children when mHSS^28^ was used (*p* = .039), whereas mSSS^27^ found no differences in age. Previous studies have reported a higher proportion of moderate‐to‐severe reactions (77%)^33^ compared to our findings of 34% (mSSS^27^) and 51% (mHSS^28^), but the need for intramuscular epinephrine in our study (20%) was similar to the previously reported need (21%).^5^

The findings that HM was tolerated in higher quantities than FM (*p* < .001, Figure S1B) and that reaction severities in FM and HM OFCs were similar (*p* = .359, Figure S1A) suggest that, excluding severe anaphylaxis, HM OFCs are generally safe to perform irrespective of the FM OFC reaction. Furthermore, even if reactions to FM emerged at small cumulative doses, HM could be tolerated in higher amounts.

These cutoff values could also be applied in health care systems that utilize an HM OFC as the initial diagnostic step, provided there are no severe anaphylactic reactions to FM in patient history. However, the cutoffs in such an unscreened population may be overly conservative, and clinical judgment is required in their interpretation. Especially, children likely to be allergic to FM could be considered for this approach, as an FM OFC could be avoided. After a positive HM OFC, use of a milk ladder, such as the Canadian milk ladder^35^ modified in our clinic to include HM and BM products on the first step, can be directly advised. Conversely, in cases of a negative HM OFC, a subsequent FM OFC should be considered to avoid delays in the reintroduction of FM into the diets of possibly non‐allergic children.

As in a previous study,^8^ we found that HM reactive children were older compared to HM tolerant children (*p* = .05), possibly reflecting a more persistent CMA phenotype. Although there was no difference in reaction severity or cumulative HM OFC dose between children aged ≤3 years and >3 years, diagnostic cutoffs appeared to have higher accuracy in younger children (Figure 4, Tables S1B,C and S2), as also shown in our previous work on FM.^25^ While larger studies specifically designed to assess the effect of age on cutoff values are needed to confirm these tentative findings, we suggest the casein sIgE cutoff value of <0.81 kU/L corresponding to 95% sensitivity can be reliably used to rule out HM allergy in ≤3‐year‐old children. For >3‐year‐old children, however, the general casein sIgE cutoff of <0.54 kU/L is recommended, as a useful 95% sensitivity cutoff could not be achieved in this age group.

This is the first study, to our knowledge, to provide casein sIgE cutoff values specifically for HM OFC outcomes. A major strength is the large cohort of children with IgE‐mediated, OFC‐confirmed CMA from a real‐life setting. The use of extensively heated milk in a rice‐based matrix allowed us to focus on the effect of heating without the confounding allergenicity‐reducing effect of a wheat matrix.^17^, ^23^, ^24^ This also enabled us to include children with frequent co‐allergies to egg and wheat.^36^ Previous studies have provided casein sIgE cutoff values for BM without requiring an FM OFC to confirm CMA.^7^, ^19^, ^31^ Thus, our study fills a significant gap in current knowledge. Furthermore, we are currently collecting data on the results of a two‐year follow‐up on the FM tolerance development rate and compliance with HM consumption. As a limitation, the low prevalence of moderate‐to‐severe reactions did not allow sufficient analysis of reaction severity predictors. Additionally, the 95% specificity cutoff values require validation in future studies, as the number of children exceeding these cutoff values was low. To build on our results, developing a logistic multivariate model to predict HM OFC results could be considered after the example recently set by Delli Colli et al.^37^ for FM OFCs.

In summary, we found that casein sIgE was the best predictor for HM OFC outcome and can be helpful in identifying children with a high or low likelihood of heated milk tolerance. A cutoff value of >14.1 kU/L provided 95% specificity and 18% sensitivity for predicting HM intolerance, while a cutoff of <0.54 kU/L predicted HM tolerance with 96% sensitivity and 46% specificity. Cutoff values were more accurate in children under 3 years of age, with casein sIgE level <0.81 kU/L serving as a reliable threshold to rule out HM allergy. HM tolerance was further associated with higher tolerated FM cumulative doses. We do not advise using SPTs in diagnostics due to their high‐resource intensity and scant additional benefit compared to sIgE. We propose the following approach for HM introduction, based on casein sIgE and clinical history. For children with low casein sIgE (<0.54 kU/L) and no history of severe reactions, we recommend home‐based introduction of HM using a milk ladder,^35^ whereas those with significant casein sensitization should first undergo an HM OFC to assess if introduction is safe. For children at high risk (e.g. those with prior severe reactions or casein sIgE >14.1 kU/L), we advise continued avoidance with reassessment within 1–2 years. Finally, HM is tolerated in higher amounts than FM, and HM OFCs are generally safe to perform irrespective of the FM OFC reaction severity.

## AUTHOR CONTRIBUTIONS

**Otso Nieminen:** Conceptualization; investigation; funding acquisition; writing – original draft; methodology; visualization; writing – review and editing; formal analysis; data curation; validation. **Kati Palosuo:** Conceptualization; writing – review and editing; methodology; investigation; project administration; supervision; resources. **Mika J. Mäkelä:** Conceptualization; funding acquisition; methodology; writing – review and editing; project administration; supervision; resources; investigation.

## FUNDING INFORMATION

This work was supported by Helsingin yliopistollinen sairaala (tutkimusrahoitus), Allergiatutkimussäätiö, Lastentautien Tutkimussäätiö, Helsingin Yliopisto, Sigrid Juséliuksen Säätiö, and Suomen Lääketieteen Säätiö.

## CONFLICT OF INTEREST STATEMENT

The authors have no conflicts of interest to declare pertaining to this article.

## PEER REVIEW

The peer review history for this article is available at https://www.webofscience.com/api/gateway/wos/peer‐review/10.1111/pai.70152.

## Supporting information


Figure S1.

Table S1.

Table S2.

Appendix S1.
