# Supplementary material for: Casein sIgE as the most accurate predictor for heated milk tolerance in Finnish children
Source: Pediatr Allergy Immunol. 2025 Jul 18;36(7):e70152. doi: 10.1111/pai.70152 (PMC12273190; doi:10.1111/pai.70152)
Supplement: Supplementary file 1 — Figure S1. [file PAI-36-e70152-s003.pdf]

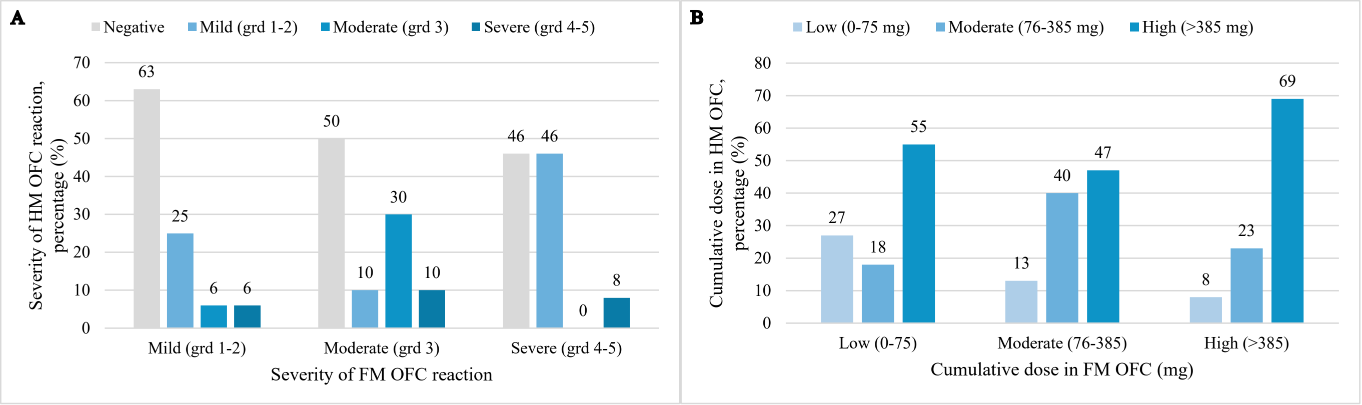

sFigure1: The effect of FM OFC reaction severity on HM OFC outcome (A, N=127) and the effect of FM OFC cumulative eliciting dose on the cumulative eliciting dose in positive HM OFCs (B, N=50). HM: heated milk, FM: fresh milk, OFC: oral food challenge, mg: milligram.
