# Supplementary material for: Casein sIgE as the most accurate predictor for heated milk tolerance in Finnish children
Source: Pediatr Allergy Immunol. 2025 Jul 18;36(7):e70152. doi: 10.1111/pai.70152 (PMC12273190; doi:10.1111/pai.70152)
Supplement: Supplementary file 2 — Table S1. [file PAI-36-e70152-s002.docx]

1A

|  | | AUC (95% CI) | Cutoff (kU/L) | Specificity (%) | Sensitivity (%) | PPV (%) | NPV (%) | LR+ | LR - | P-value |
| --- | --- | --- | --- | --- | --- | --- | --- | --- | --- | --- |
| 95% specificity cutoff | |  |  |  |  |  |  |  |  |  |
| Casein sIgE | 0.786 (0.708–0.864) | 14.1 | 94.8 | 18.0 | 69.2 | 64.0 | 3.47 | 0.86 | **<0.001*** |  |
| Milk sIgE | 0.690 (0.598–0.782) | 33.9 | 94.8 | 14.0 | 63.6 | 62.9 | 2.70 | 0.91 | **<0.001*** |  |
| Alpha-lactalbumin sIgE | 0.626 (0.523–0.728) | 9.85 | 94.8 | 28.0 | 77.8 | 67.0 | 5.39 | 0.76 | **0.017*** |  |
| Beta-lactoglobulin sIgE | 0.597 (0.497–0.696) | 20.1 | 94.8 | 2.0 | 20.0 | 59.8 | 0.39 | 1.03 | 0.066 |  |
| BSA | 0.514 (0.412–0.616) | 6.17 | 94.8 | 6.0 | 42.9 | 60.8 | 1.16 | 0.99 | 0.790 |  |
| 95% sensitivity cutoff | |  |  |  |  |  |  |  |  |  |
| Casein sIgE | 0.786 (0.708–0.864) | 0.54 | 45.5 | 96.0 | 53.3 | 94.6 | 1.76 | 0.088 | **<0.001*** |  |
| Milk sIgE | 0.690 (0.598–0.782) | 1.46 | 27.8 | 96.0 | 46.2 | 91.3 | 1.32 | 0.15 | **<0.001*** |  |
| Alpha-lactalbumin sIgE | 0.626 (0.523–0.728) | 0.03 | 5.2 | 94.0 | 39.2 | 57.1 | 0.99 | 1.16 | **0.017*** |  |
| Beta-lactoglobulin sIgE | 0.597 (0.497–0.696) | 0.07 | 14.3 | 96.0 | 42.1 | 84.6 | 1.12 | 0.28 | 0.066 |  |
| BSA | 0.514 (0.412–0.616) | NA | NA | NA | NA | NA | NA | NA | 0.790 |  |
| Optimal cutoff | |  |  |  |  |  |  |  |  |  |
| Casein sIgE | 0.786 (0.708–0.864) | 1.74 | 72.7 | 78.0 | 65.0 | 83.6 | 2.86 | 0.30 | **<0.001*** |  |
| Milk sIgE | 0.690 (0.598–0.782) | 4.92 | 59.7 | 72.0 | 53.7 | 76.7 | 1.79 | 0.47 | **<0.001*** |  |
| Alpha-lactalbumin sIgE | 0.626 (0.523–0.728) | 2.54 | 62.3 | 64.0 | 52.5 | 72.7 | 1.70 | 0.58 | **0.017*** |  |
| Beta-lactoglobulin sIgE | 0.597 (0.497–0.696) | 1.48 | 58.4 | 62.0 | 49.2 | 70.3 | 1.49 | 0.65 | 0.066 |  |
| BSA | 0.514 (0.412–0.616) | 0.01 | 36.4 | 70.0 | 41.7 | 65.1 | 1.10 | 0.83 | 0.790 |  |

1B

|  | | AUC (95% CI) | Cutoff (kU/L) | Specificity (%) | Sensitivity (%) | PPV (%) | NPV (%) | LR+ | LR - | P-value |
| --- | --- | --- | --- | --- | --- | --- | --- | --- | --- | --- |
| 95% specificity cutoff | |  |  |  |  |  |  |  |  |  |
| Casein sIgE | 0.833 (0.738–0.928) | 14.1 | 95.6 | 13.0 | 60.0 | 68.3 | 2.93 | 0.91 | **<0.001*** |  |
| Milk sIgE | 0.710 (0.582–0.838) | 20.7 | 95.6 | 21.7 | 71.4 | 70.5 | 4.89 | 0.82 | **0.005*** |  |
| Alpha-lactalbumin sIgE | 0.617 (0.467–0.767) | 8.48 | 95.6 | 17.4 | 66.7 | 69.4 | 3.91 | 0.86 | 0.117 |  |
| Beta-lactoglobulin sIgE | 0.620 (0.482–0.758) | 17.0 | 95.6 | 8.7 | 50.0 | 67.2 | 1.96 | 0.96 | 0.108 |  |
| BSA | 0.470 (0.323–0.616) | 6.68 | 95.6 | 8.7 | 50.0 | 67.2 | 1.96 | 0.96 | 0.683 |  |
| 95% sensitivity cutoff | |  |  |  |  |  |  |  |  |  |
| Casein sIgE | 0.833 (0.738–0.928) | 0.81 | 60.0 | 95.7 | 55.0 | 96.4 | 2.39 | 0.07 | **<0.001*** |  |
| Milk sIgE | 0.710 (0.582–0.838) | 1.46 | 37.8 | 95.7 | 44.0 | 94.4 | 1.54 | 0.12 | **0.005*** |  |
| Alpha-lactalbumin sIgE | 0.617 (0.467–0.767) | 0.02 | 0.0 | 95.7 | 32.8 | 0.0 | 0.96 | NA | 0.117 |  |
| Beta-lactoglobulin sIgE | 0.620 (0.482–0.758) | 0.10 | 24.4 | 95.7 | 39.3 | 91.7 | 1.27 | 0.18 | 0.108 |  |
| BSA | 0.470 (0.323–0.616) | NA | NA | NA | NA | NA | NA | NA | 0.683 |  |
| Optimal cutoff | |  |  |  |  |  |  |  |  |  |
| Casein sIgE | 0.833 (0.738–0.928) | 1.26 | 73.3 | 87.0 | 62.5 | 91.7 | 3.26 | 0.18 | **<0.001*** |  |
| Milk sIgE | 0.710 (0.582–0.838) | 7.96 | 73.3 | 60.9 | 53.8 | 78.6 | 2.28 | 0.53 | **0.005*** |  |
| Alpha-lactalbumin sIgE | 0.617 (0.467–0.767) | 1.53 | 68.9 | 65.2 | 51.7 | 79.5 | 2.10 | 0.50 | 0.117 |  |
| Beta-lactoglobulin sIgE | 0.620 (0.482–0.758) | 0.46 | 44.4 | 78.3 | 41.9 | 80.0 | 1.41 | 0.49 | 0.108 |  |
| BSA | 0.470 (0.323–0.616) | 2.93 | 93.3 | 13.0 | 50.0 | 67.7 | 1.96 | 0.93 | 0.683 |  |

1C

|  | | AUC (95% CI) | Cutoff (kU/L) | Specificity (%) | Sensitivity (%) | PPV (%) | NPV (%) | LR+ | LR - | P-value |
| --- | --- | --- | --- | --- | --- | --- | --- | --- | --- | --- |
| 95% specificity cutoff | |  |  |  |  |  |  |  |  |  |
| Casein sIgE | 0.729 (0.601–0.856) | 20.7 | 96.9 | 18.5 | 83.3 | 58.5 | 5.93 | 0.84 | **0.003*** |  |
| Milk sIgE | 0.650 (0.510–0.790) | 39.8 | 96.9 | 14.8 | 80.0 | 57.4 | 4.74 | 0.88 | **0.049*** |  |
| Alpha-lactalbumin sIgE | 0.602 (0.452–0.751) | 17.0 | 96.9 | 22.2 | 85.7 | 59.6 | 7.11 | 0.80 | 0.181 |  |
| Beta-lactoglobulin sIgE | 0.569 (0.419–0.720) | 22.3 | 96.9 | 0.0 | 0.0 | 53.4 | 0.0 | 1.03 | 0.361 |  |
| BSA | 0.537 (0.389–0.685) | 7.74 | 96.9 | 3.7 | 50.0 | 54.4 | 1.19 | 0.99 | 0.626 |  |
| 95% sensitivity cutoff | |  |  |  |  |  |  |  |  |  |
| Casein sIgE | 0.729 (0.601–0.856) | 0.33 | 31.2 | 96.3 | 54.2 | 90.9 | 1.40 | 0.12 | **0.003*** |  |
| Milk sIgE | 0.650 (0.510–0.790) | 2.59 | 34.4 | 96.3 | 55.3 | 91.7 | 1.47 | 0.11 | **0.049*** |  |
| Alpha-lactalbumin sIgE | 0.602 (0.452–0.751) | 0.03 | 3.1 | 96.3 | 45.6 | 50.0 | 0.99 | 1.19 | 0.181 |  |
| Beta-lactoglobulin sIgE | 0.569 (0.419–0.720) | 0.07 | 6.2 | 96.3 | 46.4 | 66.7 | 1.03 | 0.59 | 0.361 |  |
| BSA | 0.537 (0.389–0.685) | NA | NA | NA | NA | NA | NA | NA | 0.626 |  |
| Optimal cutoff | |  |  |  |  |  |  |  |  |  |
| Casein sIgE | 0.729 (0.601–0.856) | 1.83 | 65.6 | 77.8 | 65.6 | 77.8 | 2.26 | 0.34 | **0.003*** |  |
| Milk sIgE | 0.650 (0.510–0.790) | 2.59 | 34.4 | 96.3 | 55.3 | 91.7 | 1.47 | 0.11 | **0.049*** |  |
| Alpha-lactalbumin sIgE | 0.602 (0.452–0.751) | 11.6 | 93.7 | 37.0 | 83.3 | 63.8 | 5.93 | 0.67 | 0.181 |  |
| Beta-lactoglobulin sIgE | 0.569 (0.419–0.720) | 1.48 | 59.4 | 70.4 | 59.4 | 70.4 | 1.73 | 0.50 | 0.361 |  |
| BSA | 0.537 (0.389–0.685) | 0.01 | 28.1 | 85.2 | 50.0 | 69.2 | 1.19 | 0.53 | 0.626 |  |

Supplementary table 1. Cutoff values for all measured sIgE tests for all children (1A, N=127), <3-year-old children (1B, N=68) and >3-year-old children (1C, N=59) to predict HM OFC outcome. Cutoff points closest to 95% specificity and 95% sensitivity were determined and optimal cutoff values were chosen based on maximal Youden’s index. For BSA, 95% sensitivity cutoff value could not be reached. sIgE: specific IgE, BSA: bovine serum albumin, AUC: area under the curve, CI: confidence interval, kU/L: kilounits per liter, PPV: positive predictive value, NPV: negative predictive value, LR+: positive likelihood ratio, LR–: negative likelihood ratio, HM OFC: heated milk oral food challenge. * denotes a statistically significant difference (p < .05) in sIgE values between HM OFC negative and positive groups.
