# Supplementary material for: Casein sIgE as the most accurate predictor for heated milk tolerance in Finnish children
Source: Pediatr Allergy Immunol. 2025 Jul 18;36(7):e70152. doi: 10.1111/pai.70152 (PMC12273190; doi:10.1111/pai.70152)
Supplement: Supplementary file 3 — Table S2. [file PAI-36-e70152-s004.docx]

A

|  | | AUC (95% CI) | Cutoff (mm) | Specificity (%) | Sensitivity (%) | PPV (%) | NPV (%) | LR+ | LR - | P-value |
| --- | --- | --- | --- | --- | --- | --- | --- | --- | --- | --- |
| 95% specificity cutoff | |  |  |  |  |  |  |  |  |  |
| FM SPT | 0.587 (0.442–0.732) | 13.5 | 95.0 | 4.3 | 33.3 | 63.3 | 0.87 | 1.01 | 0.253 |  |
| HM SPT | 0.839 (0.729–0.949) | 11.0 | 95.0 | 4.3 | 33.3 | 63.3 | 0.87 | 1.01 | **<0.001*** |  |
| 95% sensitivity cutoff | |  |  |  |  |  |  |  |  |  |
| FM SPT | 0.587 (0.442–0.732) | 4.5 | 10.0 | 95.7 | 37.9 | 80.0 | 1.06 | 0.43 | 0.253 |  |
| HM SPT | 0.839 (0.729–0.949) | 4.5 | 47.5 | 95.7 | 51.2 | 95.0 | 1.82 | 0.09 | **<0.001*** |  |
| Optimal cutoff | |  |  |  |  |  |  |  |  |  |
| FM SPT | 0.587 (0.442–0.732) | 7.5 | 57.5 | 60.9 | 45.2 | 71.9 | 1.43 | 0.68 | 0.253 |  |
| HM SPT | 0.839 (0.729–0.949) | 5.5 | 77.5 | 87.0 | 69.0 | 91.2 | 3.86 | 0.17 | **<0.001*** |  |

B

|  | | AUC (95% CI) | Cutoff (mm) | Specificity (%) | Sensitivity (%) | PPV (%) | NPV (%) | LR+ | LR - | P-value |
| --- | --- | --- | --- | --- | --- | --- | --- | --- | --- | --- |
| 95% specificity cutoff | |  |  |  |  |  |  |  |  |  |
| FM SPT | 0.644 (0.499–0.790) | 14.5 | 96.9 | 14.8 | 80.0 | 57.4 | 4.74 | 0.88 | 0.058 |  |
| HM SPT | 0.667 (0.524–0.810) | 11.5 | 96.9 | 7.4 | 66.7 | 55.4 | 2.37 | 0.96 | **0.028*** |  |
| 95% sensitivity cutoff | |  |  |  |  |  |  |  |  |  |
| FM SPT | 0.644 (0.499–0.790) | 4.5 | 6.2 | 96.3 | 46.4 | 66.7 | 1.03 | 0.59 | 0.058 |  |
| HM SPT | 0.667 (0.524–0.810) | 3.5 | 9.4 | 88.9 | 45.3 | 50.0 | 0.98 | 1.19 | **0.028*** |  |
| Optimal cutoff | |  |  |  |  |  |  |  |  |  |
| FM SPT | 0.644 (0.499–0.790) | 9.5 | 81.2 | 51.9 | 70.0 | 66.7 | 2.77 | 0.59 | 0.058 |  |
| HM SPT | 0.667 (0.524–0.810) | 7.5 | 87.5 | 48.1 | 76.5 | 66.7 | 3.85 | 0.59 | **0.028*** |  |

Supplementary table 2. Age-specific cutoff values for HM SPT and FM SPT to predict HM OFC outcome for < 3-year-old children (A, N=63) and >3-year-old children (B, N=59). Cutoff points closest to 95% specificity and 95% sensitivity were determined and optimal cutoff values were chosen based on maximal Youden’s index. For >3-year-old children, 95% sensitivity could not be reached for HM SPT. FM: fresh milk, HM: heated milk, SPT: skin prick test, OFC: oral food challenge, AUC: area under the curve, CI: confidence interval, mm: millimeter, PPV: positive predictive value, NPV: negative predictive value, LR+: positive likelihood ratio, LR–: negative likelihood ratio. * denotes a statistically significant (p < .05) difference.
