# Supplementary material for: Casein sIgE as the most accurate predictor for heated milk tolerance in Finnish children
Source: Pediatr Allergy Immunol. 2025 Jul 18;36(7):e70152. doi: 10.1111/pai.70152 (PMC12273190; doi:10.1111/pai.70152)
Supplement: Supplementary file 4 — Appendix S1. [file PAI-36-e70152-s001.docx]

**Rice porridge recipe used in heated milk oral food challenges**

**Ingredients**:

5 L of reduced-fat milk (contains lactose)

8 dL of porridge rice

2 tsp. of salt

Dairy-free margarine for oiling the oven tray

**Cooking instructions:**

Oil the oven tray and mix all the ingredients together. Heat in the oven in 175 **°**C for 90 minutes.
